# Uremic Toxins Induce Kidney Fibrosis by Activating Intrarenal Renin–Angiotensin–Aldosterone System Associated Epithelial-to-Mesenchymal Transition

**DOI:** 10.1371/journal.pone.0034026

**Published:** 2012-03-30

**Authors:** Chiao-Yin Sun, Shih-Chung Chang, Mai-Szu Wu

**Affiliations:** 1 School of Medicine, Chang Gung University, Taoyuan, Taiwan; 2 Division of Nephrology, Chang Gung Memorial Hospital, Keelung, Taiwan; 3 Department of Biochemical Science and Technology, National Taiwan University, Taipei, Taiwan; University of Tokushima, Japan

## Abstract

**Background:**

Uremic toxins are considered to have a determinant pathological role in the progression of chronic kidney disease. The aim of this study was to define the putative pathological roles of the renal renin–angiotensin–aldosterone system (RAAS) and renal tubular epithelial-to-mesenchymal transition (EMT) in kidney fibrosis induced by (indoxyl sulfate) IS and (*p*-cresol sulfate) PCS.

**Methods:**

Mouse proximal renal tubular cells (PKSV-PRs) treated with IS or PCS were used. Half-nephrectomized B-6 mice were treated with IS or PCS for 4 weeks. In the losartan treatment study, the study animal was administrated with IS+losartan or PCS+losartan for 4 weeks.

**Results:**

IS and PCS significantly activated the intrarenal RAAS by increasing renin, angiotensinogen, and angiotensin 1 (AT1) receptor expression, and decreasing AT2 receptor expression *in vitro* and *in vivo*. IS and PCS significantly increased transforming growth factor-β1 (TGF-β1) expression and activated the TGF-β pathway by increasing Smad2/Smad2-P, Smad3/Smad3-P, and Smad4 expression. The expression of the EMT-associated transcription factor Snail was increased by IS and PCS treatment. IS and PCS induced the phenotype of EMT-like transition in renal tubules by increasing the expression of fibronectin and α-smooth muscle actin and decreasing the expression of E-cadherin. Losartan significantly attenuated the expression of TGF-β1 and Snail, and decreased kidney fibrosis induced by IS and PCS in vivo.

**Conclusion:**

Activating the renal RAAS/TGF-β pathway has an important pathological role in chronic kidney injury caused by IS and PCS. IS and PCS may increase Snail expression and induce EMT-like transition.

## Introduction

Chronic kidney disease is a progressive disease. The renal toxicity of uremic toxins is thought to have a determinant pathological role in the progression of chronic kidney disease. Indoxyl sulfate (IS) and *p*-cresol sulfate (PCS) are important examples of the protein-bound uremic toxins that are not dialyzable. They have similar features, such as the albumin-binding site, and both originate from protein fermentation [1], [2]. These molecules have been linked to oxidative injury [3], [4]. IS and PCS have been clinically associated with the risk of cardiovascular disease [5], [6]. Our previous clinical study had showed that serum levels of IS and PCS are significantly associated with the progression of chronic kidney disease [7]. Accumulated evidence has revealed that IS and PCS play significant pathological roles in chronic kidney injury *in vitro* and *in vivo* [8]–[10].

Activation of the renin–angiotensin–aldosterone system (RAAS) is one of the main mechanisms of chronic ischemic nephropathy-associated progression of chronic kidney disease. Inhibition of the RAAS is currently one of the most powerful therapies for slowing the progression of chronic kidney disease [11], [12]. RAAS activation could further activate transforming growth factor-β (TGF-β), which is known as an important fibrogenic cytokine in the development of kidney fibrosis [13], [14]. IS stimulates renal synthesis of TGF-β1 and the progression of renal failure *in vivo* [15].

Epithelial-to-mesenchymal transition (EMT) is considered an important mechanism in the development and progression of malignancies [16], [17]. EMT induced by TGF-β1 has been reported as a possible mechanism for kidney fibrosis [18], [19]. In addition, RAAS activation is able to induce EMT by both TGF-dependent and TGF-independent actions, both *in vitro* and *in vivo* [20]

In this study, we tested the hypothesis that IS and PCS could induce kidney fibrosis by activating the intrarenal RAAS and inducing renal tubular EMT. In addition, the putative pathological role of EMT in kidney fibrosis induced by IS and PCS was studied.

## Materials and Methods

### Ethics Statement

All animal experiments were approved by the local committee for experimental animals at the Chang Gung Memorial Hospital (permit number: 2010121406).

### Mouse proximal renal tubular cell culture

Mouse proximal renal tubular cells (PKSV-PRs), provided by Dr. Alain Vandewalle, were cultured in a modified culture medium as described previously [21], [22]. Serum starvation was conducted for 24 h to synchronize the cells prior to IS (Sigma-Aldrich, St. Louis, MO, USA) and PCS (Kureha Corporation, Tokyo, Japan) treatment. During the IS and PCS treatment course, serum-free medium was used. The duration of the treatment was 72 h. The concentrations of IS and PCS used in the *in vitro* experiments are described in the figures and figure legends. The control PKSV cells were treated with the same volume of phosphate buffered saline (PBS) and cultured under the same conditions. In the losartan (Sigma-Aldrich, St. Louis, MO, USA) treatment study, PKSV cells after 24 h serum starvation were treated with losartan and IS/PCS for 72 h. The dosage for IS, PCS and losartan were illustrated in figures. Each treatment was repeated in triplet for the following analysis.

### Animal study

Ten-week-old male B-6 mice with ½-nephrectomy were used in this study. The study animals were divided into control, experiment, and treatment groups. The control mice (n = 8) received daily PBS injections at the same volume as the experimental mice for 4 weeks. The experimental mice received intraperitoneal injection with IS (n = 8) or PCS (n = 8) at a dose of 100 mg/kg/day for 4 weeks. There were 2 subgroups of treatment mice. The IS+losartan group received intraperitoneal injection with IS (100 mg/kg/day) and oral losartan treatment (25 mg/kg/day) via a feeding tube for 4 weeks (n = 8). The PCS+losartan group received intraperitoneal injection with PCS (100 mg/kg/day) and oral losartan treatment (25 mg/kg/day) via a feeding tube for 4 weeks (n = 8). The renal cortex was microdissected for further analysis at the end of the animal study.

### Real-time polymerase chain reaction

Whole kidney or cell extracts (10 mg) were used to isolate total RNA using a commercial kit (RNeasy Kit; Qiagen, Hilden, Germany) according to the manufacturer's instructions, including DNase treatment. Five micrograms of total RNA was then reverse transcribed using reverse transcriptase (Bio-Rad, Berkeley, CA, USA) with random primers. Real-time polymerase chain reaction (PCR) was performed in 25 µL SYBR Green PCR Master Mix (Applied Biosystems, Carlsbad, CA, USA) containing 0.6 mol/L primers and 1 µg cDNA by using an iQ5 real-time PCR detection system (Bio-Rad, Berkeley, CA, USA). The thermal cycling program and PCR primers are listed in Table S1. Each PCR reaction was performed in triplicate, and the mean Ct value was used for statistical analysis. Messenger RNA expression was standardized to β-actin expression levels, followed by normalization to the control group.

### Western blotting analysis

Kidney tissues and PKSV cells were homogenized, and total protein was extracted using a commercial kit according to the manufacturer's instructions (Protein Extraction Kit; Millipore, Billerica, MA, USA). Kidney and cell extracts (30 µg protein per lane) were mixed with a sample loading buffer and separated on 12% sodium dodecyl sulfate-polyacrylamide gel. Proteins were electrotransferred onto polyvinylidene fluoride membranes (0.2 µm; Immun-Blot; Bio-Rad, Berkeley, CA, USA). The antibodies used for western blotting are listed in Table S2. The intensity of each band was quantified using the NIH Image software, and the densitometric intensity corresponding to each band was normalized against β-actin expression.

### Assays for angiotensin II and transforming growth factor-β1

The angiotensin II and TGF-β1 concentrations in the culture medium were analyzed by EIA (Angiotensin II EIA Kit; RayBiotech, Inc., Norcross, GA, USA) and ELISA (TGF-beta 1 Quantikine ELISA Kit; R&D Systems, Inc., Minneapolis, MN, USA) methods respectively. The analysis was performed according to the product instruction, and read by SpectraMax M3 (Molecular Devices, Inc., Sunnyvale, CA, USA). Each test was repeated in triplet, and the average value was used for the following statistical analysis.

### Immunofluorescence staining

Cryostat sections of renal tissue and PKSV cells fixed with ice-cold acetone were incubated overnight at 4°C with primary antibodies against mouse; this was followed by incubation with appropriate secondary immunofluorescent antibodies for 1 h at room temperature. The antibodies for immunofluorescence staining and the folds of dilution are described in Supplement 2. The cell nucleus was stained with Hoechst stains (Molecular Probes, Eugene, OR, USA).

### Histopathological examination

Sections of paraffin-embedded specimens were stained with the Masson trichrome to assess the degree of renal fibrosis. Four random and non-overlapping sections from each kidney tissue were examined under high power field (400×). The nephrosclerosis scores including interstitial and glomerular fibrosis were analyzed. Glomerular and interstitial fibrosis was assessed, and the severity of nephrosclerosis was semiquantified with a 0 to 3+ scale (0: no lesions present; 1: mild; 2: moderate; 3: severe) for both interstitial and glomerular fibrosis.

### Statistical analysis

All data are expressed as mean ± SE. Data of *in vitro* studies was compared with ANOVA with post hoc test. Data from different study groups *in vivo* were compared using the Wilcoxon–Mann–Whitney test, and *P* values of <0.05 were considered statistically significant.

## Results

### IS and PCS activated intrarenal RAAS

**Figure 1:**
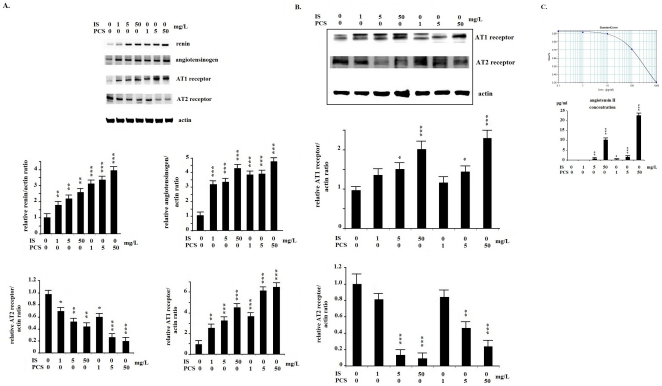
Indoxyl sulfate and *p*-cresol sulfate activated the renin–angiotensin–aldosterone system in vitro. A: Real-time polymerase chain reaction analysis shows that mouse proximal renal tubular cells (PKSV) cells had significantly increased renin, angiotensinogen, and angiotensin 1 (AT1) receptor mRNA expression, and decreased AT2 receptor mRNA expression. B: Western blotting shows that PKSV cells treated with indoxyl sulfate (IS) and *p*-cresol sulfate (PCS) at concentrations of 5 and 50 mg/L had significantly increased AT1 receptor protein expression. IS and PCS at concentrations of 1, 5, and 50 mg/L significantly decreased AT2 receptor protein expression *in vitro*. C: The angiotensin II concentrations in the culture medium were measured by EIA method. The angiotensin II concentration of control PKSV cells was lower than detection limit. Treatment with IS (5 and 50 mg/L) and PCS (1, 5 and 50 mg/L) significantly increased angiotensin II production. (*: *P*<0.05; **: *P*<0.01; ***: *P*<0.001, *vs.* control).

**Figure 2:**
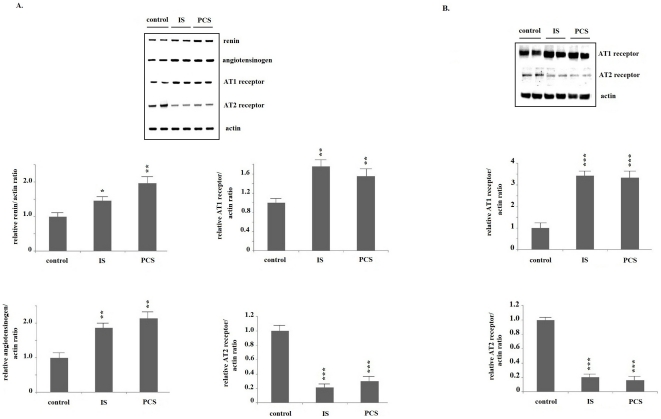
Indoxyl sulfate and *p*-cresol sulfate activated renin–angiotensin–aldosterone system in vivo. A: Real-time polymerase chain reaction analysis show that mice treated with indoxyl sulfate (IS) and *p*-cresol sulfate (PCS) had significantly increased intra-renal renin, angiotensinogen, and angiotensin 1 (AT1) receptor mRNA expression, and decreased AT2 receptor mRNA expression. B: Western blotting shows that mice treated with IS and PCS had significantly increased intrarenal AT1 receptor and decreased AT2 receptor protein expression. (*: *P*<0.05; **: *P*<0.01; ***: *P*<0.001, *vs.* control).

PKSV cells treated with IS or PCS had significantly increased renin, angiotensinogen, and angiotensin 1 (AT1) receptor mRNA expression, as revealed by real-time PCR assay. The mRNA expression of the AT2 receptor was significantly decreased in PKSV cells treated with IS or PCS (Figure 1A). Western blotting showed that PKSV cells treated with IS and PCS at concentrations of 5 and 50 mg/L had significantly increased AT1 receptor protein expression. IS and PCS at concentrations of 5 and 50 mg/L significantly decreased AT2 receptor protein expression *in vitro* (Figure 1B). The concentrations of angiotensin II in the culture medium were shown in the Figure 1C. The mice undergoing chronic IS or PCS treatment had significantly increased intrarenal renin, angiotensinogen, and AT1 receptor mRNA expression, as determined by real-time PCR assay. The mRNA expression of the AT2 receptor was significantly decreased in mice treated with IS or PCS than in the negative controls (Figure 2A). The results of western blotting showed that, compared with the negative control mice, the mice treated with IS or PCS had significantly increased AT1 receptor and decreased AT2 receptor expression (Figure 2B).

### IS and PCS activated the TGF-β pathway and increased expression of the EMT-associated transcription factor Snail

**Figure 3:**
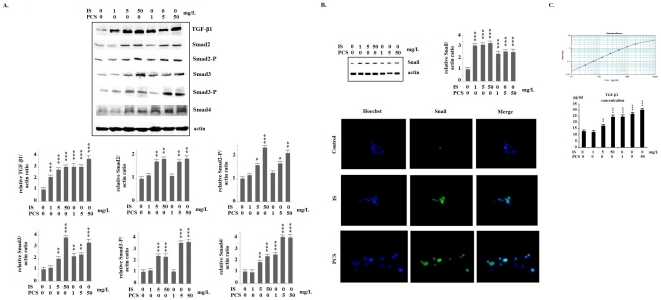
Indoxyl sulfate and *p*-cresol sulfate activated transforming growth factor-β pathway and increased Snail expression in vitro. A: Results of western blotting show that mouse proximal renal tubular cells (PKSV) cells had significantly increased protein expression of Sma- and Mad-related protein (Smad)2/Smad2-P, Smad3/Smad3-P, and Smad4, when treated with indoxyl sulfate (IS) and *p*-cresol sulfate (PCS). B: Western blotting and immunostaining for Snail show that PKSV cells had significantly increased nuclolear expression of Snail, when treated with IS and PCS. In the immunostaining study of Snail, PKSV cells were treated with 5 mg/dL IS or PCS for 3 days. C: The transforming growth factor-β1 (TGF-β1) concentrations in the culture medium were measured by ELISA method. PKSV cells treated with IS (5 and 50 mg/L) and PCS (1, 5 and 50 mg/L) had significantly higher TGF-β1 concentration in culture medium than control cells. (600×) (*: *P*<0.05; **: *P*<0.01; ***: *P*<0.001, *vs.* control).

**Figure 4:**
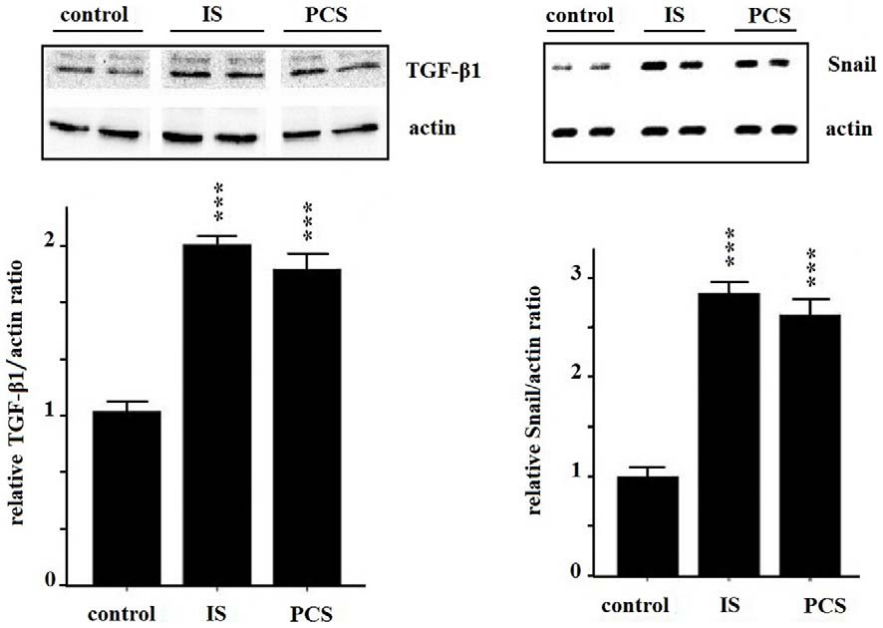
Indoxyl sulfate and *p*-cresol sulfate increased tumor growth factor-β1 and Snail protein expression in vivo. Western blot results show that mice treated with indoxyl sulfate and *p*-cresol sulfate had significantly increased intrarenal transforming growth factor-β1 and Snail protein expression. (*: *P*<0.05; **: *P*<0.01; ***: *P*<0.001, *vs.* control).

Western blotting analysis of cultured PKSV cells showed that IS and PCS significantly increased TGF-β1 expression. The expression of downstream signals of the TGF-β pathway, such as Sma- and Mad-related protein (Smad)2/Smad2-P, Smad3/Smad3-P, and Smad4, were also increased significantly *in vitro* (Figure 3A). In addition, the expression of the EMT-associated transcription factor Snail was increased by IS and PCS treatment. The results of western blotting and immunostaining for Snail are shown in Figure 3B. The TGF-β1 concentrations in the culture medium were shown in the Figure 3C. The animal study revealed that chronic IS and PCS injection could significantly increase renal TGF-β1 and Snail expression *in vivo* (Figure 4).

### IS and PCS induced EMT-like transition

**Figure 5:**
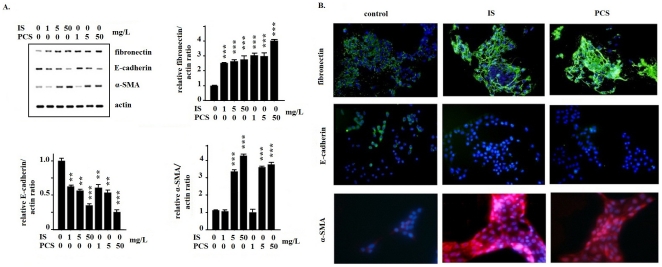
Indoxyl sulfate and *p*-cresol sulfate induced epithelial-to-mesenchymal-like transition in mouse proximal renal tubular cells. A: Real-time polymerase chain recation results show that mouse proximal renal tubular (PKSV) cells treated with indoxyl sulfate (IS) and *p*-cresol sulfate (PCS) had significantly increased fibronectin and decreased E-cadherin expression. PKSV cells treated with IS and PCS at concentrations of 5 and 50 mg/L had significantly increased α-smooth muscle actin (SMA) expression. B: Results of immunohistological staining show that PKSV cells treated with IS and PCS at a concentration of 5 mg/L for 3 days had significant staining for fibronectin and α-SMA. The staining for E-cadherin was significantly decreased in PKSV cells treated with IS or PCS. (400×) (*: *P*<0.05; **: *P*<0.01; ***: *P*<0.001, *vs.* control).

The results of real-time PCR showed that PKSV cells treated with IS or PCS at concentrations of 1, 5, and 50 mg/L significantly increased fibronectin and decreased E-cadherin mRNA expression. The α-smooth muscle actin (SMA) expression was significantly increased in PKSV cells treated with IS or PCS at concentrations of 5 and 50 mg/L (Figure 5A). The results of immunostaining for fibronectin, E-cadherin, and α-SMA *in vitro* are shown in Figure 5B. Positive staining for fibronectin and α-SMA were significantly increased in PKSV cells treated with IS or PCS at a concentration of 5 mg/L. Staining for E-cadherin was significantly decreased in PKSV cells treated with IS or PCS than in the control group.

**Figure 6:**
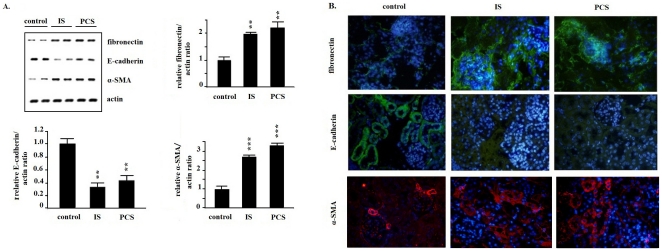
Indoxyl sulfate and *p*-cresol sulfate induced epithelial-to-mesenchymal-like transition in vivo. A: Real-time polymearse chain reaction results show that mice treated with indoxyl sulfate (IS) and *p*-cresol sulfate (PCS) had significantly increased fibronectin and α- smooth muscle actin (SMA) expression in kidney. E-cadherin expression was significantly decreased in mice treated with IS and PCS. B: Results of immunohistological staining show that the staining for fibronectin and α-SMA was significantly increased in mice treated with IS and PCS. The staining for E-cadherin was significantly decreased in mice treated with IS and PCS. (400×) (*: *P*<0.05; **: *P*<0.01; ***: *P*<0.001, *vs.* control).

The animal study results show that chronic treatment with IS or PCS could significantly increase renal fibronectin and α-SMA mRNA expression. IS and PCS significantly decreased renal E-cadherin mRNA expression *in vivo* (Figure 6A). The results of immunostaining in study animals also revealed that chronic IS or PCS treatment significantly increased the interstitial expression of fibronectin and α-SMA and decreased E-cadherin expression (Figure 6B).

### RAAS inhibition decreased kidney fibrosis induced by IS and PCS

**Figure 7:**
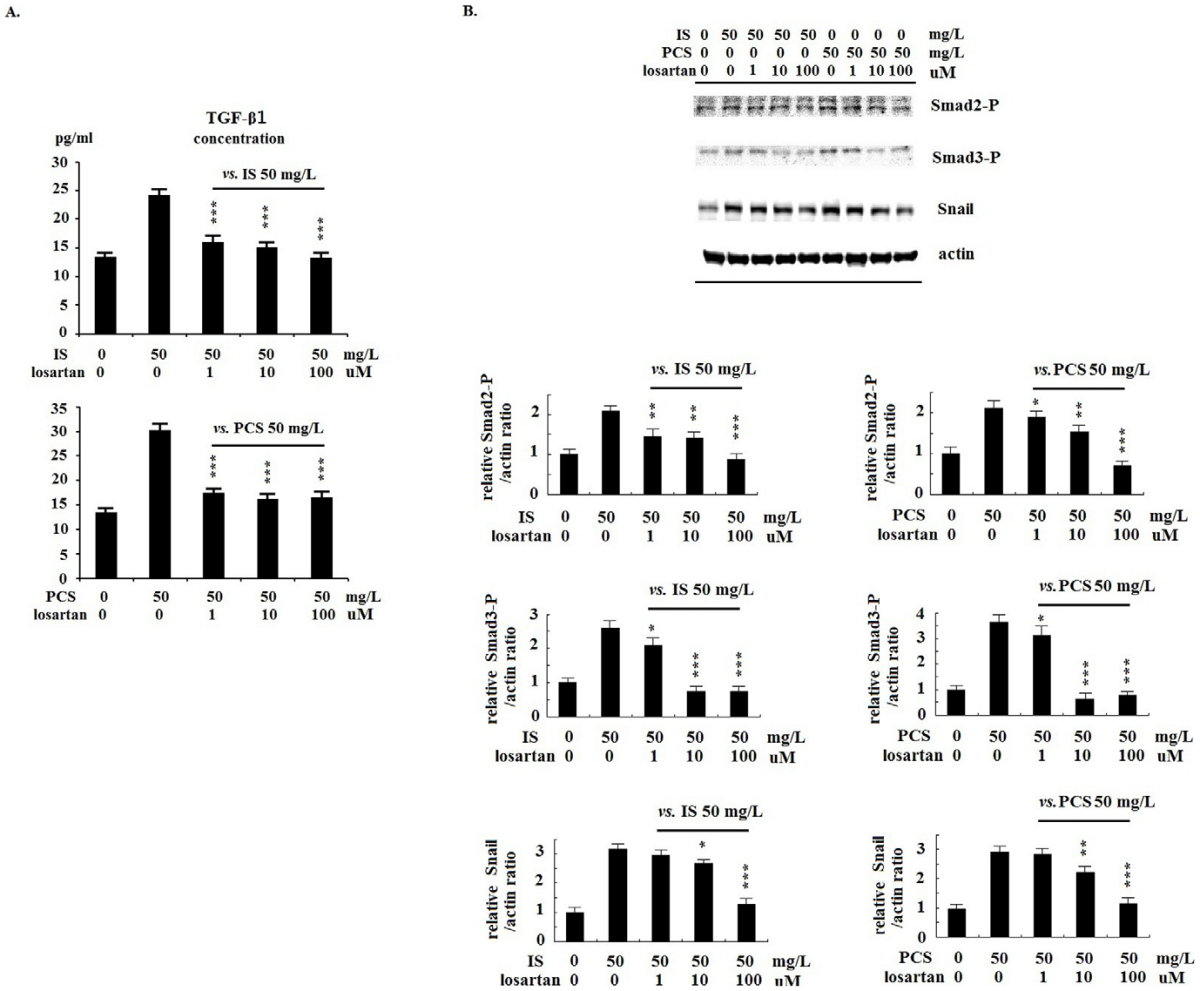
Renin–angiotensin–aldosterone system inhibition with losartan significantly inhibited transforming growth factor-β1/Smads pathway and decreased Snail expression in vitro. A: ELISA analysis showed that losartan (1, 10, and 100 uM) significantly decreased the transforming growth factor-β1 concentration in the culture medium of PKSV cells treated with indoxyl sulfate (IS) (50 mg/L) and *p*-cresol sulfate (PCS) (50 mg/L). In addition, the Westren blotting results showed that significantly decreased the expression of Smad2-P, Smad3-P and Snail in PKSV cells treated wioth IS and PCS. (*: *P*<0.05; **: *P*<0.01; ***: *P*<0.001).

**Figure 8:**
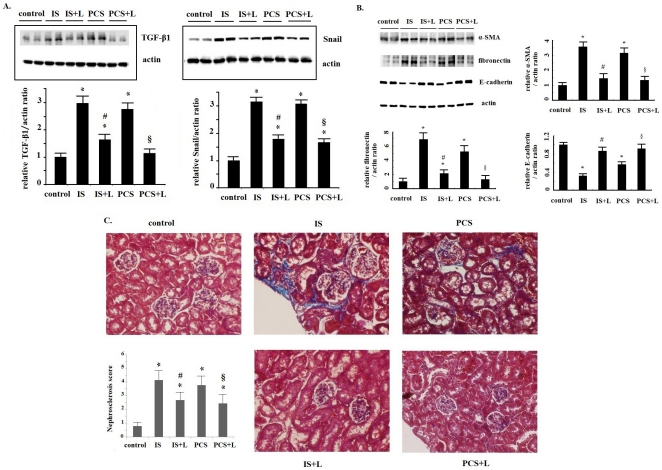
Renin–angiotensin–aldosterone system inhibition with losartan significantly decreased transforming growth factor-β1 and Snail expression and kidney injury in vivo. A: Western bloting shows that losartan significantly decreased renal transforming growth factor-β1 and Snail expression in mice treated with indoxyl sulfate (IS) and *p*-cresol sulfate (PCS). B: Western blotting results revealed that losartan significantly increaesd E-cadherin, and decreased fibronectin and α- smooth muscle actin protein expression in mice treated with IS or PCS. C: Masson trichrome staining results revealed that kidneys of mice treated with IS and PCS had significantly increased intersitial and glomerular fibrosis than control mice. Renin–angiotensin–aldosterone system inhibition with losartan significantly decreased kidney fibrosis in mice treated with IS and PCS. D: The nephrosclerosis scores of control and experimental mice were plotted. Nephrosclerosis scores were significantly higher in the IS- and PCS-injected mice than in the control mice. The nephrosclerosis scores were significantly lower in the losartan treatment groups (IS+L and PCS+L) than in the IS and PCS groups. (400×) (*: *P*<0.05, *vs.* control; #: *P*<0.05, IS+L *vs*. IS; §: *P*<0.05, PCS+L *vs.* PCS) (L: losartan).

The results of ELISA analysis showed that losartan treatment (1, 10 and 100 uM) significantly decreased the TGF-β1 concentrations in the culture medium of PKSV cells treated with IS and PCS (50 mg/L) (Figure 7A). Losartan treatment also decreased the Smad2-P, Smad3-P and Snail expression *in vitro* by Western blotting (Figure 7B). We performed a further animal study involving RAAS blockage with losartan. The results of western blotting showed significant decrease in renal TGF-β1 and Snail expression in mice treated with losartan than in the untreated controls (Figure 8A). In addition, losartan treatment significantly increased E-cadherin, and decreased fibronectin and α-SMA protein expression in mice injected with IS or PCS (Figure 8B). Comparing the mice treated with IS or PCS only, losartan treatment significantly decreased kidney fibrosis induced by IS and PCS (Figure 8C). The nephrosclerosis scores for the study animals are plotted in Figure 8C. Compared with the control mice, the mice treated with IS and PCS had significantly higher nephrosclerosis scores (IS *vs.* control: 4.19±0.76 *vs.* 0.89±0.36, *P*<0.05; PCS *vs.* control: 3.84±0.58 *vs.* 0.89±0.36, *P*<0.05). Mice treated with losartan had significantly lower nephrosclerosis scores than those treated with IS or PCS only (IS+losartan *vs.* IS: 2.66±0.53 *vs.* 4.19±0.76, *P*<0.05; PCS+losartan: *vs.* PCS: 2.44±0.47 *vs.* 3.84±0.58, *P*<0.05).

## Discussion

The main findings of this study suggest that IS and PCS might activate the intrarenal RAAS and TGF/Smad pathway. EMT-like transition of tubular cells activated by the EMT-associated transcription factor Snail might be a possible mechanism for kidney fibrosis induced by IS and PCS.

The main pathophysiological mechanisms associated with chronic kidney disease result from the activation of the RAAS. Oxidative stress leads to activation of the RAAS, with a subsequent increase in the levels of angiotensin II and TGF-β1, which are 2 important molecular mediators of kidney injury [23]. Previous studies using IS have demonstrated that IS increases renal oxidative stress, induces abnormal oxygen consumption in renal tubules, and aggravates chronic hypoxia of the kidney [24]. Our results show that both IS and PCS could activate renal RAAS, increasing renin, angiotensinogen, and AT1 receptor expression and decreasing AT2 receptor expression *in vitro* and *in vivo*. In addition, our data demonstrate that blocking RAAS with losartan could significantly decrease the severity of nephrosclerosis induced by IS and PCS.

Accumulated evidence has demonstrated that TGF-β plays an important role in the progression of renal disease. Smad2/3 activation is critical for the pro-fibrotic effect of TGF-β [25]. Smad4 binds to Smad2/3 and facilitates the translocation of the heteromeric complex into the nucleus [26]. Previous studies have revealed that IS stimulates TGF-β1 synthesis in proximal tubular cells and the progression of renal failure. IS also increases renal TIMP-1 and pro-alpha 1(I) collagen expression in uremic rats [15], [27]. Activation of the RAAS is a key mediator in the progression of renal disease [23]. Many profibrotic effects of RAAS activation are mediated by stimulation of TGF-β. Components of RAAS activation including renin, angiotensin II/III and aldosterone all upregulate TGF-β expression [28]. There is accumulating evidence that angiotensin II is able to induce EMT by both TGF-β-dependent and TGF-β-independent actions, both *in vitro* and *in vivo* [29], [30]. Previous study has showed that blocking TGF-β-dependent pathway could significantly decrease chronic kidney fibrosis by inhibiting EMT [18], [31]. Our study has revealed that both IS and PCS could increase renal TGF-β1 expression *in vitro* and *in vivo*. In addition, our *in vitro* study has shown that IS and PCS activate the TGF-β pathway by activating the Smad2/3 pathway and increasing Smad4 expression. Blocking the RAAS with losartan could significantly decrease renal TGF-β1 expression in mice injected with IS or PCS. The results of this study suggested that activations of TGF-β-dependent signaling pathway by RAAS might have important roles in the kidney injury by IS and PCS.

EMT is a process by which differentiated epithelial cells undergo a phenotypic conversion that gives rise to matrix-producing fibroblasts and myofibroblasts. Tubular cell EMT is considered to be a fundamental contributor to renal fibrosis [20]. TGF-β/Smad, integrin-linked kinase, and Wnt/β-catenin signaling are essential intracellular signal transduction pathways for controlling the process of EMT [32]. TGF-β1 is the most potent factor for EMT, and many other prosclerotic factors have indirect effects on EMT, via the induction of TGF-β1 [33], [34]. RAAS activation is able to induce EMT by both TGF-dependent and TGF-independent actions [20]. Angiotensin II mediates EMT in tubular cells by activating ANG 1–7/MAS-1-dependent pathways [35]. Our results show that IS and PCS could induce EMT in tubular cells *in vitro* and *in vivo*, and activation of the TGF-β/Smad pathway by RAAS is a possible mechanism.

Our study also shows that IS and PCS could increase the expression of the EMT-associated transcription factor Snail in renal tubules. Snail has been reported to be a key effector in cancer-related EMT, which is associated with malignant tumor progression [36]. The interaction between the Snail and TGF-β signaling pathway is mutual. It was reported that TGF-β1 hyper-expression could increase Snail expression [37], [38]. Previous studies have showed that Snail was a key mediator in the TGF-β1-mediated EMT. Blocking Snail function could mitigate the TGF-β1-mediated EMT [39]. In breast cancer, Snail and Slug could activate TGF-β signaling pathway [40]. Snail induction by adriamycin is also known to cause mesenchymal conversion of podocytes [41]. A previous study with obstructive nephropathy (UUO) animal model revealed that blocking RAAS with angiotensin II receptor, renin receptor blockers or both could decrease the Snail-1 and TGF-β1 expression, and attenuated UUO-related kidney fibrosis [42]. Our study also showed similar results. It was suggested that an increase in Snail expression as a result of TGF-β pathway activation has an important role in the EMT process induced by IS and PCS in renal tubular cells.

**Figure 9:**
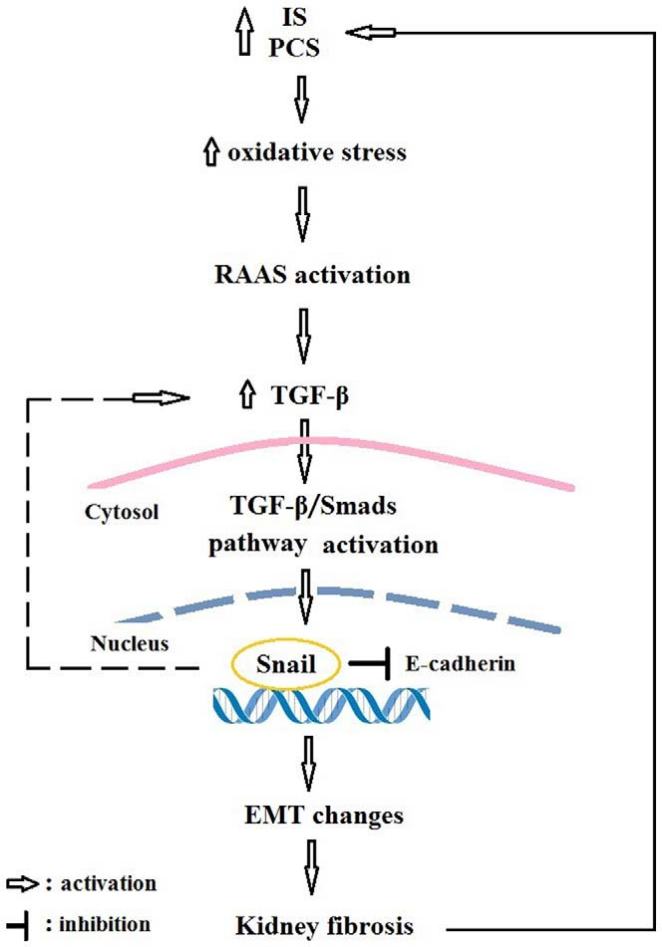
Potential mechanisms for the vitreous cycle of kidney injury induced by indoxyl sulfate and *p*-cresol sulfate. IS, indoxyl sulfate; PCS, *p*-cresol sulfate; RAAS, renin-angiotension-aldosterone system; TGF-β, transforming growth factor-β; EMT, epithelial-to-mesenchymal transition.

The putative mechanisms for the kidney fibrosis induced by IS and PCS was summarized in Figure 9. The activation of the renal RAAS/TGF-β pathway has an important pathological role in chronic kidney injury caused by IS and PCS. IS and PCS may increase Snail expression which might further increase TGF-β expression, and induce EMT-like transition in renal tubular cells. Our study details the pathological mechanisms of chronic renal injury caused by uremic toxins. However, it should be stressed that this study has some limitations because only a mouse model and a cultured mouse renal tubular cell line were used. Whether these results can extend to primary culture renal tubular cells, and be applied to human disease, remains to be determined.

## Supporting Information

Table S1 **Primers for quantitative PCR (5′à3′).** The thermal cycling program comprises an initial denature step at 95°C for 10 minutes, followed by 95°C for 15 seconds and 65°C for 1 minute for 40 cycles.
(DOC)

Table S2 **Antibodies for western blotting and immunostaining.**
(DOC)
