# Supplementary material for: Uremic Toxins Induce Kidney Fibrosis by Activating Intrarenal Renin–Angiotensin–Aldosterone System Associated Epithelial-to-Mesenchymal Transition
Source: PLoS One. 2012 Mar 30;7(3):e34026. doi: 10.1371/journal.pone.0034026 (PMC3316590; doi:10.1371/journal.pone.0034026)
Supplement: Table S1 — Primers for quantitative PCR (5′à3′). The thermal cycling program comprises an initial denature step at 95°C for 10 minutes, followed by 95°C for 15 seconds and 65°C for 1 minute for 40 cycles. (DOC) [file pone.0034026.s001.doc]

**Table S1:**

**Primers for quantitative PCR (5’🡪3’).** The thermal cycling program comprises an

initial denature step at 95 ^o^C for 10 minutes, followed by 95 ^o^C for 15 seconds and

65^o^C for 1 minute for 40 cycles.

|  | Forward | Backward |
| --- | --- | --- |
| Renin | TCT GGG CAC TCT TGT TGC TC | GGG GGA GGT AAG ATT GGT CAA |
| Angiotensinogen | GTA CAG ACA GCA CCC TAC TT | CAC GTC ACG GAG AAG TTG TT |
| AT1 receptor | TCG CTA CCT GGC CAT TGT C | TGA CTT TGG CCA CCA GCA T |
| AT2 receptor | CCT TCT TGG ATG CTC TGA CC | GCG GTT TCC AAC AAA ACA AT |
| Fibronectin | ACA GAA ATG ACC ATT GAA GG | TGT CTG GAG AAA GGT TGA TT |
| α-SMA | CAG GCA TGG ATG GCA TCA ATC AC | ACT CTA GCT GTG AAG TCA GTG TCG |
| E-cadherin | AAT GGC GGC AAT GCA ATC CCA AGA | TGC CAC AGA CCG ATT GTG GAG ATA |
| β-actin | CAC ACT GTG CCC ATC TAC G | GCC ATC TCT TGC TCG AAG TC |
