# Supplementary material for: Uremic Toxins Induce Kidney Fibrosis by Activating Intrarenal Renin–Angiotensin–Aldosterone System Associated Epithelial-to-Mesenchymal Transition
Source: PLoS One. 2012 Mar 30;7(3):e34026. doi: 10.1371/journal.pone.0034026 (PMC3316590; doi:10.1371/journal.pone.0034026)
Supplement: Table S2 — Antibodies for western blotting and immunostaining. (DOC) [file pone.0034026.s002.doc]

**Table S2:**

**Antibodies for western blotting and immunostaining.**

| Antibody for  western blot | Source | Dilution |
| --- | --- | --- |
| anti-AT1 receptor | Abcam Plc. | 500x |
| anti-AT2 receptor | Abcam Plc. | 500x |
| anti-snail | Cell Signaling, Inc. | 500x |
| anti-TGF-β1 | Abcam Plc. | 500x |
| anti-E-cadherin | Cell Signaling, Inc. | 500x |
| anti-α-SMA | Abcam Plc. | 500x |
| anti-fibronectin | Abcam Plc. | 500x |
| anti-β-actin | Abcam Plc. | 1000x |

| Antibody for immunostaining | Source | Dilution |
| --- | --- | --- |
| anti-snail | Cell Signaling, Inc. | 100x |
| anti-fibronectin | Abcam Plc. | 100x |
| anti-α-SMA | Abcam Plc. | 100x |
| anti-E-cadherin | Cell Signaling, Inc. | 100x |
